# Increased level of RAB39B leads to neuronal dysfunction and behavioural changes in mice

**DOI:** 10.1111/jcmm.17704

**Published:** 2023-03-28

**Authors:** Zijie Wang, Mengxi Niu, Naizhen Zheng, Jian Meng, Yiru Jiang, Dingting Yang, Peijie Yao, Tingting Yao, Hong Luo, Huaxi Xu, Yunlong Ge, Yun‐wu Zhang, Xian Zhang

**Affiliations:** ^1^ Center for Brain Sciences, the First Affiliated Hospital of Xiamen University Institute of Neuroscience, Fujian Provincial Key Laboratory of Neurodegenerative Disease and Aging Research School of Medicine Xiamen University Xiamen China; ^2^ Department of Neurosurgery Xiang'an Hospital of Xiamen University Xiamen China

**Keywords:** autophagy, mouse behaviours, neuronal morphology, RAB39B, synaptic transmission, X‐linked intellectual disability

## Abstract

Duplications of the Xq28 region are a common cause of X‐linked intellectual disability (XLID). The *RAB39B* gene locates in Xq28 and has been implicated in disease pathogenesis. However, whether increased dosage of RAB39B leads to cognitive impairment and synaptic dysfunction remains elusive. Herein, we overexpressed RAB39B in mouse brain by injecting AAVs into bilateral ventricles of neonatal animals. We found that at 2 months of age, neuronal overexpression of RAB39B impaired the recognition memory and the short‐term working memory in mice and resulted in certain autism‐like behaviours, including social novelty defect and repetitive grooming behaviour in female mice. Moreover, overexpression of RAB39B decreased dendritic arborization of primary neurons in vitro and reduced synaptic transmission in female mice. Neuronal overexpression of RAB39B also altered autophagy without affecting levels and PSD distribution of synaptic proteins. Our results demonstrate that overexpression of RAB39B compromises normal neuronal development, thereby resulting in dysfunctional synaptic transmission and certain intellectual disability and behavioural abnormalities in mice. These findings identify a molecular mechanism underlying XLID with increased copy numbers of Xq28 and provide potential strategies for disease intervention.

## 1 INTRODUCTION

X‐linked intellectual disability (XLID) consists of a wide range of disorders that are characterized by cognitive impairment and decreased adaptability.^1^, ^2^, ^3^ XLID is caused by mutations in X chromosome genes, and both deletions and duplications on the X chromosome make important contributions to disease phenotypes.^4^, ^5^ So far mutations in more than 140 X chromosome genes, including *RAB39B*, have been associated with XLID.^6^

The *RAB39B* gene encodes a small GTP enzyme protein that participates in vesicular membrane trafficking in eukaryotic cells.^7^, ^8^ Loss‐of‐function mutations in the *RAB39B* gene have been associated with XLID, Parkinson's disease, autism spectrum disorders (ASDs), epileptic seizure and macrocephaly.^9^, ^10^, ^11^ Recent studies including ours have found that RAB39B regulates the PI3K‐AKT–mTOR pathway and that RAB39B deficiency impairs neuronal morphology and functions and leads to abnormal behaviours resemble those found in patients with *RAB39B* loss‐of‐function mutations.^9^, ^12^, ^13^, ^14^

In addition to RAB39B deficiency, excessive RAB39B may also cause XLID.^15^ The XLID syndrome involving *RAB39B* duplications occurs from ~0.5 Mb Xq28 subregional replication, which is mediated by nonallelic homologous recombination between low copy repeats (LCRs) intron 22 homologous regions 1 (int22h1) and 2 (int22h2).^16^, ^17^, ^18^, ^19^, ^20^ Int22h1/int22h2‐mediated Xq28 duplication results in a novel recognizable XLID syndrome characterized by intellectual disability, neurobehavioural abnormalities, recurrent infections and facial deformity.^18^, ^19^ Both *RAB39B* and *CLIC2* genes are consistently contained in the smallest overlapping region in patients with int22h1/int22h2‐mediated Xq28 duplication, implying that increased dosage of the two genes may be responsible for disease phenotypes.^18^, ^19^, ^21^ However, the underlying molecular mechanism remains largely elusive and the contribution of excessive RAB39B to XLID has yet to be confirmed in animal models.

## 2 MATERIALS AND METHODS

### 2.1 Animals

C57BL/6 wild‐type mice were obtained from Xiamen University Laboratory Animal Center. Mice were housed 3–5 per cage in the animal facility with a 12 h light/dark cycle and had free access to food and water. All procedures and protocols involving animals were performed in accordance with the guidelines of the National Institutes of Health Guide for the Care and Use of Laboratory Animals and were approved by the Animal Ethics Committee of Xiamen University.

### 2.2 Intracerebroventricular injection of adeno‐associated virus (AAV)

For neuronal overexpression of RAB39B, a pAAV‐SYN‐EGFP‐P2A‐3FLAG‐RAB39B construct expressing full‐length human RAB39B was generated and packaged into AAV (serotype 2/9, OBIO Technology). One μl RAB39B‐expressing AAV or its control AAV (8 × 10^12^ viral genomes/mL) was slowly injected into each ventricle of postpartum day 0 (P0) wild‐type mice as described previously.^22^, ^23^, ^24^

### 2.3 Primary neuron cultures and AAV infection

Mouse hippocampi were isolated from P0 mice and were digested with trypsin at 37°C for 20 min. Tissues were centrifuged for 5 min at 1500 rpm and resuspended in DMEM media with 10% FBS. The primary neurons were plated on poly‐L‐lysine (Sigma‐Aldrich) coated dishes and cultured in neurobasal media (Gibco) supplemented with 2% B‐27 (Gibco), 2 mM L‐glutamine (Gibco), and 1% penicillin–streptomycin (Gibco) in a 5% CO_2_ incubator at 37°C. Culture media were changed every 3 days. Cultured primary neurons were infected with AAV on DIV 3. Immunofluorescence staining was performed on DIV 12.

### 2.4 Quantitative real‐time PCR (qRT‐PCR)

To compare the expression of exogenous *RAB39B* and endogenous *Rab39b*, total RNAs were extracted using the TRIzol Reagent (Invitrogen). Equal amounts of RNA were reverse‐transcribed using the ReverTra Ace qPCR RT Kit (TOYOBO). qRT‐PCR was performed on the LightCycler 480 II instrument (Roche) with FastStart Universal SYBR Green Master (ROX) (Roche). A pair of primers that amplify both human *RAB39B* and mouse *Rab39b* was used. Primers sequences were as follows:

**Table:** 

human *RAB39B* and mouse *Rab39b*:
forward primer: 5′‐ATCGAGCCAGGAAAACGCAT‐3′;
reverse primer: 5′‐GTAGTAGGCGCGAGTGATGG‐3′;
*β‐actin*:
forward primer: 5′‐AGCCATGTACGTAGCCATCCA‐3′;
reverse primer: 5′‐TCTCCGGAGTCCATCACAATG‐3′.

### 2.5 Immunofluorescence staining

Mice were anaesthetised and perfused transcardially with PBS. Whole brain was quickly dissected and fixed in 4% paraformaldehyde at 4°C for 24 h and then dehydrated in 30% sucrose. Brain tissues were embedded in OCT and 30‐μm‐thick sections were collected with a cryostat microtome (Leica). Mouse brain sections were permeabilized and blocked in block buffer (0.2% Triton X‐100 and 5% goat serum in PBS) at room temperature for 1 h and then incubated with primary antibodies against NeuN (Abcam, ab177487, 1:200), GFAP (Proteintech, 16825‐1‐AP, 1:200) or FLAG (ABclonal, AE092, 1:100) overnight at 4°C and with an appropriate fluorescence‐conjugated secondary antibody (Thermo Fisher Scientific, A11012, 1:400) for 1 h at room temperature. The sections were washed in PBS and stained with DAPI (Sigma‐Aldrich, D9542, 1:1000) for 10 min. Z‐stack images were captured using an A1R (Nikon) confocal microscope or an Aperio Versa 200 (Leica) microscope.

Primary neurons were fixed in 4% paraformaldehyde and permeabilized in PBS containing 0.2% Triton X‐100 and then blocked in 2% BSA at room temperature for 1 h. Samples were incubated with a primary antibody against MAP2 (CST, 4542 S, 1:200) overnight at 4°C, incubated with an appropriate fluorescence‐conjugated secondary antibody (Thermo Fisher Scientific, A11012, 1:400) at room temperature for 1 h and then stained with DAPI for 10 min. Z‐stack images were obtained by an A1R (Nikon) confocal microscope. Sholl analysis was performed using the ImageJ software (NIH). Neurite branches were tracked and reconstructed by Simple Neurite Tracer plugin. The dendritic arborizations were quantified by the number of branches intersecting a group of concentric circles with an interval of 10 μm drawn with the cell body as the centre.

### 2.6 Efficiency of AAV infection in vivo

To determine the efficiency of AAV in vivo, EGFP and NeuN fluorescent images were obtained from brain slices using an A1R (Nikon) confocal microscope with a 20× objective. One brain section from each mouse showing the same layer of the hippocampal CA3, cerebral cortical L5, and mPFC regions was used for co‐localization analysis. The numbers of EGFP‐ and/or NeuN‐positive cells were counted manually for comparison. Neuronal transduction rate of AAV‐RAB39B was calculated by dividing the number of NeuN‐positive cells expressing EGFP by the total number of NeuN‐positive cells from z‐stack projections of the aforementioned brain regions.

### 2.7 Electrophysiology

Electrophysiology was recorded as described previously.^25^ Briefly, 3‐month‐old mice were anaesthetised with isoflurane and brains were quickly dissected and transferred to an ice‐cold solution (64 mM NaCl, 2.5 mM KCl, 1.25 mM NaH_2_PO_4_, 10 mM MgSO_4_, 0.5 mM CaCl_2_, 26 mM NaHCO_3_, 10 mM glucose, 120 mM sucrose, pH 7.4, 290–320 mOsm). 400‐μm‐thick brain slices were prepared by a Leica VT1200S vibrating microtome. Slices were recovered at 32°C for 1 h and incubated in artificial cerebrospinal fluid (ACSF: 126 mM NaCl, 3.5 mM KCl, 1.25 mM NaH_2_PO_4_, 1.3 mM MgSO_4_, 2.5 mM CaCl_2_, 11 mM NaHCO_3_ and 10 mM glucose, pH 7.4, 290–300 mOsm) for at least 1 h at room temperature. All solutions were saturated with 95% O_2_ /5% CO_2_ (volume/volume). Tetrodotoxin (1 μM) was added to perfusing ACSF to block sodium channels. Glass pipettes filled with solution containing 140 mM CsCH_3_SO_3_, 2 mM MgCl_2_·6H_2_O, 5 mM TEA‐Cl, 10 mM HEPES, 1 mM EGTA, 2.5 mM Mg‐ATP and 0.3 mM Na‐GTP, pH 7.3, 280 mOsm were placed in CA1 neurons. Miniature excitatory postsynaptic currents (mEPSCs) and miniature inhibitory postsynaptic currents (mIPSCs) were recorded at a holding potential of −70 mV and 0 mV, respectively. The resistance of pipettes was 5–8 MΩ. Data were filtered at 2 kHz and sampled at 10 kHz.

### 2.8 Preparation of synaptosome and PSD fractions

Synaptosome and PSD fractions from mouse brain were prepared as described previously.^26^ Briefly, mouse brain tissues were homogenized in cold sucrose buffer (0.32 M sucrose and 25 mM HEPES, pH 7.4). The homogenates were centrifuged at 1400 *g* for 10 min to separate the supernatant (S1) from the nuclei and large debris fraction. The S1 fraction was then centrifuged at 10,000 *g* for 12 min. The resulted precipitation (P2; crude synaptosomal fraction) was washed twice with sucrose buffer and then resuspended in cold HBS buffer (25 mM HEPES, pH 7.4, and 150 mM NaCl) to obtain synaptosome fraction. The synaptosome fraction was suspended in HBS buffer with 1% Triton at 4°C for 30 min, and the PSD fraction was obtained after centrifugation at 40,000 *g* for 30 min.

### 2.9 Immunoblot analysis

Mouse brain tissues were homogenized and lysed in RIPA lysis buffer (150 mM NaCl, 25 mM Tris–HCl, pH 7.5, 0.1% sodium dodecyl sulfate, and 1% Nonidet P‐40) supplemented with the Complete Protease and Phosphatase Inhibitor Cocktail (Roche). Protein concentration was determined using the Pierce BCA Protein Assay (Thermo Fisher Scientific). Equal amounts of protein lysates were separated by SDS‐polyacrylamide gel electrophoresis and transferred to polyvinylidene fluoride membrane. Membrane was blocked with 5% skim milk and incubated first with indicated primary antibodies and then with appropriate horseradish peroxidase (HRP)‐conjugated secondary antibodies. Protein band intensity was quantified using ImageJ.

Antibodies used were anti‐RAB39B (D‐12162‐1‐AP), anti‐GluA2 (11994‐1‐AP), anti‐GluN2A (19953‐1‐AP), anti‐GluN2B (19954‐1‐AP) and anti‐SYN1 (20258‐1‐AP) from Proteintech; anti‐GluN1 (5704 s), anti‐PSD95 (3450 s), anti‐β‐actin (8457 s), anti‐p62 (5114 s), anti‐LC3B (3868 s), anti‐mTOR (2983 S), anti‐phosphorylated mTOR (Ser2448) (5536 S), anti‐Akt (9272 S), anti‐phosphorylated Akt (Ser473) (9271 S), anti‐S6 (2217 s) and anti‐phosphorylated S6 (Ser240/244) (5364 s) from Cell Signaling Technology; anti‐GluA1 (04–855), anti‐GluA3 (MAB5416) and anti‐VGluT1 (MAB5502) from Millipore; and anti‐synaptophysin (SYP) (S5768) from Sigma‐Aldrich. HRP‐conjugated goat anti‐mouse (31430) and goat anti‐rabbit (31460) IgG (H + L) secondary antibodies were from Thermo Fisher Scientific.

### 2.10 Behavioural tests

Behavioural tests were carried out at 2 months of age. Mice were given 30 min of adaptation to the experimental environment before test every day. The experimental environment was soundproof. The apparatuses were cleaned with 75% ethanol between two experiments. Behavioural tests were recorded and analysed using the Smart 3.0 video tracking system (Panlab, Harvard Apparatus) unless otherwise described.

### 2.11 T/Y‐maze tests

T/Y‐maze tests were used to evaluate the spontaneous alternating behaviour and thus short‐term working memory of mice as described previously.^27^ T/Y maze has three unanimous arms (30 cm (L) × 6 cm (W) × 15 cm (H)), showing a ‘T’ / ‘Y’ shape. Mice were placed in the centre of the maze and allowed to explore freely for 5 min. Alternation triplet (%) was calculated and used for comparison.

### 2.12 Novel object recognition test

Novel object recognition test was used to evaluate the impairment of recognition memory in mice. On the first day, mice were allowed to acclimate in an open field box (40 cm (L) × 40 cm (W) × 40 cm (H)) for 10 min. The next day, mice were placed in the box with two identical objects (A and B) and allowed to explore freely for 10 min. On the third day, one old object (B) was replaced with a novel object (C), and mice were allowed to explore in the box for 10 min. The time spent to explore the novel object (C) and the familiar object (A) was measured and calculated as the recognition index (RI = T_novel_/(T_novel_ + T_familiar_)) for comparison as described previously.^28^

### 2.13 Three‐chamber social interaction test

The device for three‐chamber social interaction test is a box containing three rectangular chambers, each of which is (20 cm (L) × 42 cm (W) × 22 cm (H)). These chambers are separated by transparent plastic plates and connected by channels. Each of the two side chambers has a metal cage large enough to hold a mouse. The three‐chamber social interaction test was performed as described previously.^29^ In the adaptation phase, testing mice were placed in the middle chamber and allowed to explore freely in the three chambers for 10 min. In the first test phase, a strange mouse (S1) was placed in the metal cage in the left chamber, whereas the cage in the right chamber was kept empty (E). Testing mice were then allowed to explore freely in the three chambers for 10 min. In the second test phase, another strange mouse (S2) of the same species was placed into the empty cage, and then testing mice were allowed to freely explore for 10 min. 2‐month‐old wild‐type mice of the same sex as the testing mice were used as social stimuli mice (S1 and S2). The sniffing time to the metal cage in each side chamber was recorded and compared.

### 2.14 Morris water maze test

The device of the Morris water maze is a circular pool filled with opaque water at 22°C. Identifiable and contrastive shapes are provided as reference clues in the four directions of the pool. A transparent platform (diameter 10 cm) is placed at 1 cm under water in the target quadrant. In the training stage, mice were placed into water from different water entry points (east, south, west and west) and given 1 min to let them swim to find and climb up to the platform every day, with the order of water entry points different every day. Each mouse was tested twice a day for 6 days. If a mouse failed to find the platform within 1 min, it was guided to the platform and stayed there for 10 s. Mouse latency to the platform was recorded. On the 7th day, the platform was removed. Mice were then put into water from the opposite quadrant to let swim for 1 min; and the time spent in each quadrant was recorded.

### 2.15 Fear conditioning test

Mice were placed in a soundproof square chamber with an electrifiable grid floor and allowed to explore the chamber freely for 120 s. Subsequently, an 80 db white noise appeared for 30 s as a conditioned stimulus (CS), and a 0.5 mA foot shock was given to mice within the last 2 s of the sound as an unconditioned stimulus (US). The appearance of CS‐US pairing was repeated three times at 1‐min intervals. After the last foot shock, mice were kept undisturbed in the room for 90 s and then removed. The % of freezing time during the 120 s of acclimation were measured as baseline for the context test. After 24 h, mice were subjected to the context test, for which mice were placed in the same chamber and allowed to freely explore the chamber for 300 s without the presence of CS and US. After another 24 h, the cue test was performed. Mice were allowed to explore freely in a new chamber with a different context for 6 min and were presented with a CS at the fourth minute. The % of freezing time during the first 180 s were measured as baseline for cue test. The freezing responses of mice were recorded and compared.

### 2.16 Open field test

Mice were placed in the centre of an open field box (40 × 40 × 40 cm) and allowed to freely explore for 10 min. Time spent in the centre and total travel distance in the arena were measured and compared.

### 2.17 High elevated plus maze test

High elevated plus maze is composed of two open arms (30 cm (L) × 6 cm (W)) and two closed arms (30 cm (L) × 6 cm (W) × 15 cm (H)). Mice were placed in the maze centre and allowed to explore freely for 5 min. The time spent on the open arm was recorded for comparison.

### 2.18 Light–dark transition test

The light–dark box (40 cm (L) × 15 cm (W) × 15 cm (H)) consists of two equally sized light and dark chambers. Mice were placed in the centre with heads facing the black chamber. Mice were allowed to explore freely for 10 min. Time spent and total distance travelled in each chamber were measured.

### 2.19 Self‐grooming test

Mice were placed individually in a clean cage with fresh bedding and were recorded by video for 15 min. Total time spent on self‐grooming during the last 10 min was scored manually. Researchers were blind to mouse genotype during scoring.

### 2.20 Marble‐burying test

Mice were placed individually in a cage filled with 5‐cm‐thick bedding and 20 marbles arranged on its surface. After 30 min, the number of marbles with at least 50% of their body buried was counted for comparison.

### 2.21 Statistical analysis

Statistical analysis was performed using the Prism 8 software (GraphPad). Data represent mean ± standard error of means (SEM). For two group comparisons, Student's *t*‐test was used. For multiple group comparisons, anova followed by appropriate post hoc tests was used. Specific test used for each comparison was also described in the figure legend. *p* < 0.05 was considered to be statistically significant.

## 3 RESULTS

### 3.1 Neuronal overexpression of RAB39B affects mouse behaviours

**FIGURE 1:**
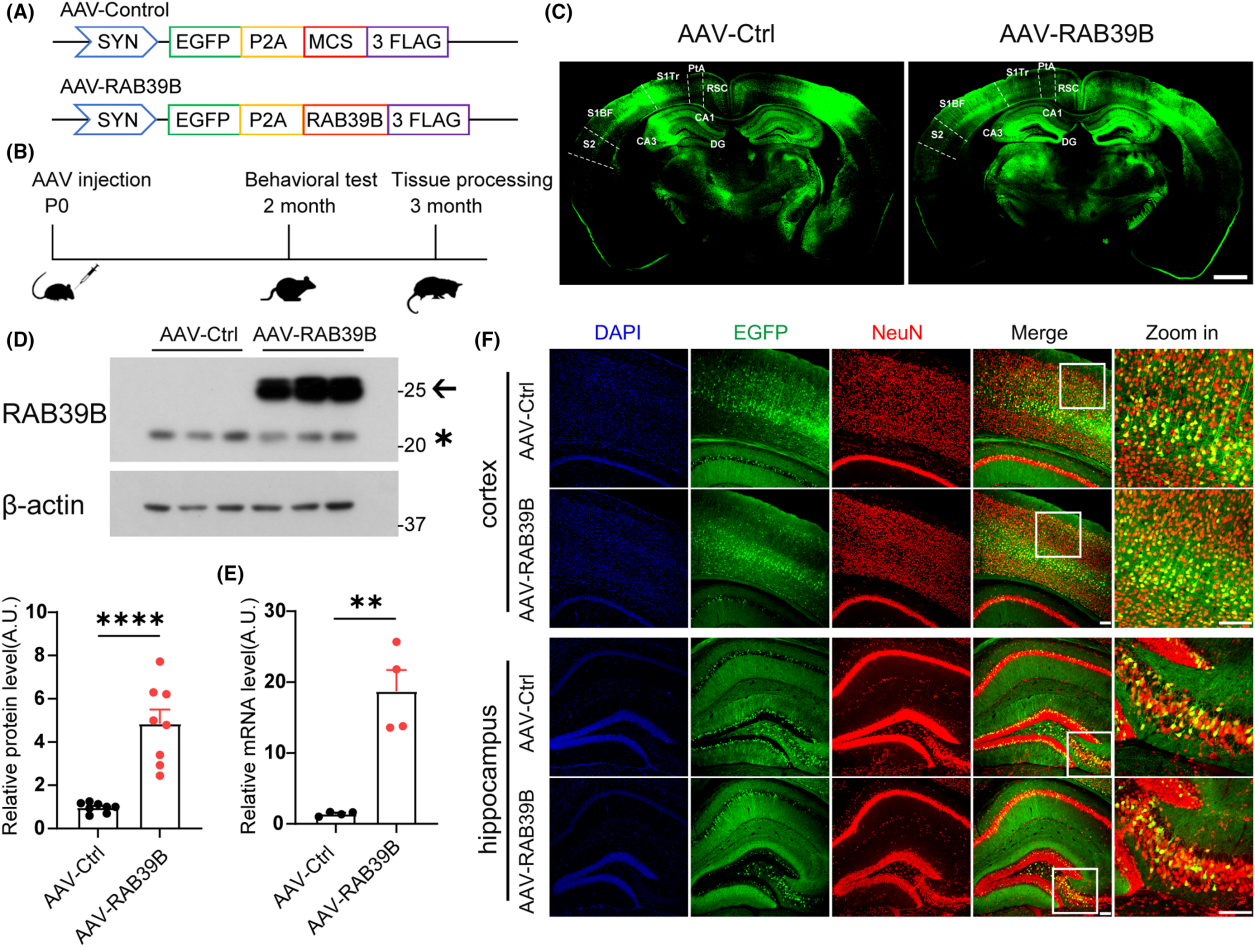
Neuronal overexpression of RAB39B in the brain of mice. (A) The schematic diagram of AAV constructs expressing EGFP control (AAV‐Control) or RAB39B (AAV‐RAB39B). (B) The workflow for AAV injection and subsequent analysis. (C) Representative images of EGFP expression in mice with AAV‐Control (Ctrl) and AAV‐RAB39B injection. Scale bar, 1000 μm. DG: dentate gyrus; RSC: retrosplenial cortex; PtA: parietal association cortex; S1Tr: primary somatosensory cortex, trunk region; S1BF: primary somatosensory cortex, barrel field; S2: secondary somatosensory cortex. (D) Western blotting for endogenous (*) and exogenous (←) RAB39B proteins in brain tissue lysates of RAB39B‐overexpressing and control mice. Endogenous and exogenous RAB39B protein levels were quantified for comparison. *n* = 8 mice per group. (E) Mouse *Rab39b* and human *RAB39B* mRNA levels in mouse brain tissues were measured by qRT‐PCR for comparison. *n* = 4 mice per group. (F) Representative images of NeuN (red) and DAPI (blue) immunostaining of cortical and hippocampal regions of 3‐month‐old mouse brain after injection with AAV‐Control and AAV‐RAB39B (represented by EGFP in green). Scale bars, 50 μm. Data represent mean ± SEM, **p < 0.01, ****p < 0.0001, Unpaired *t*‐test.

To ascertain whether overexpression of RAB39B participates in XLID, we injected AAVs overexpressing RAB39B or AAV‐EGFP (as a control) into bilateral ventricles of neonatal C57BL/6 wild‐type mice, carried out behavioural tests at 2 months of age and acquired brain tissues for further analysis at 3 months of age (Figure 1A,B). EGFP fluorescence observation demonstrated that multiple brain regions including cortex, hippocampus, prefrontal cortex (PFC), striatum and amygdala were infected with AAVs (Figures 1C and S1A–C). Immunostaining using an anti‐FLAG antibody also confirmed that FLAG‐tagged exogenous RAB39B was expressed in multiple regions of the brain (Figure S1D). Immunoblotting of mouse brain tissues indicated that the expression of exogenous RAB39B was about 5 folds of that of endogenous RAB39B (Figure 1D). While qRT‐PCR analysis indicated that exogenous *RAB39B* expression was about 12 folds of endogenous *Rab39b* expression (Figure 1 E). Moreover, we found that EGFP expression colocalized predominantly with NeuN‐ but not GFAP‐positive cells in cortical, hippocampal and mPFC regions, suggesting that exogenous RAB39B was specifically expressed in neurons. The percentage of infected neurons in each region was comparable (Figures 1F and S2A,B).

Treated mice were subjected to behavioural tests at 2 months of age. In open field tests, neither male nor female mice with neuronal overexpression of RAB39B showed differences from respective controls in their time spent in the central region and their total travel distance (Figure S3A), implying that neuronal overexpression of RAB39B has no effect on mouse anxiety. Consistently, in high elevated plus maze tests (Figure S3B) and light–dark transition tests (Figure S3C), neuronal overexpression of RAB39B did not alter the anxiety and locomotor activity of both male and female mice.

**FIGURE 2:**
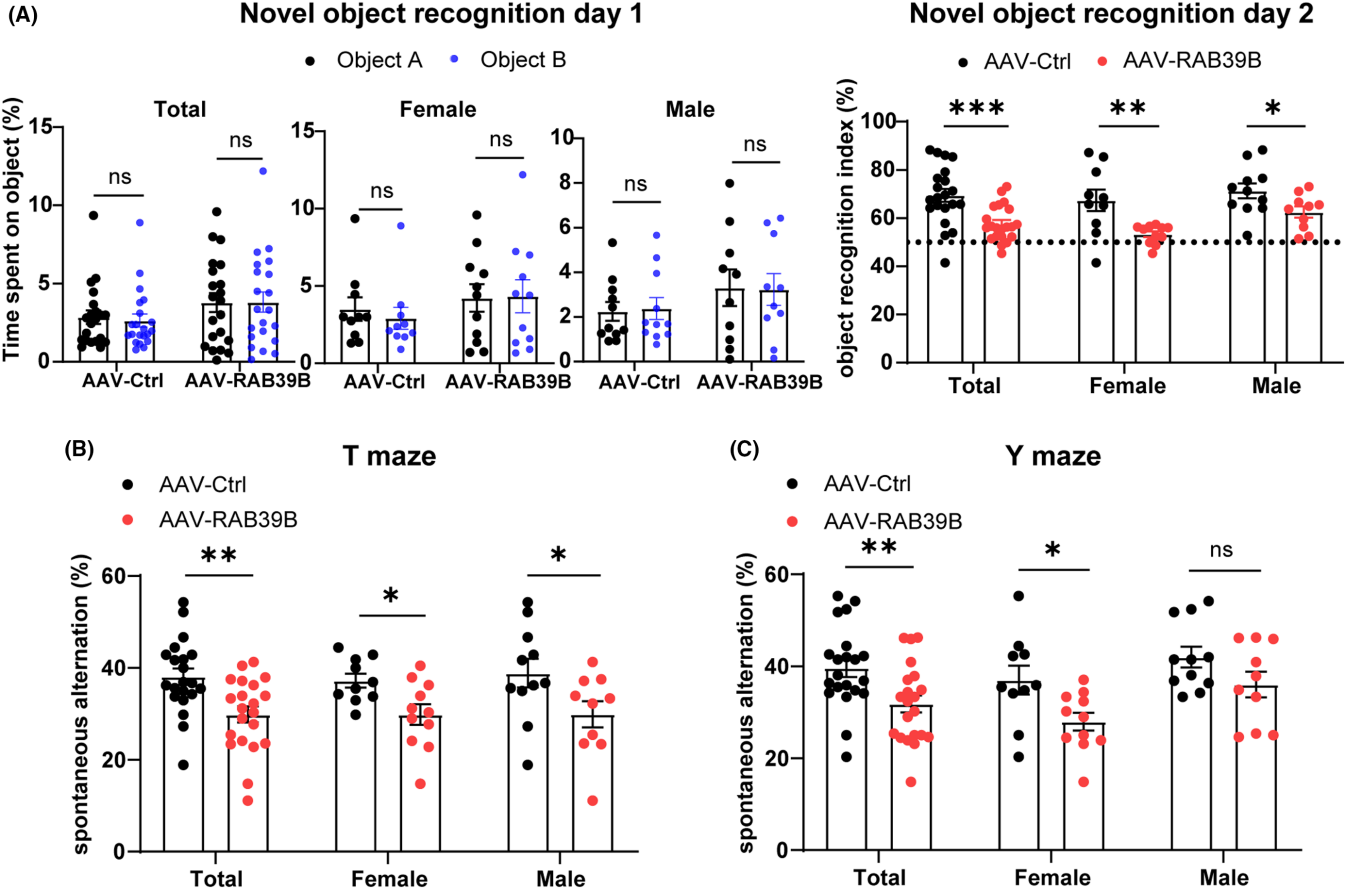
Neuronal overexpression of RAB39B impairs recognition and short‐term working memory in mice. (A) In novel object recognition tests, the recognition capability of two identical objects A and B by mice (males only, females only and total with males and females combined) were evaluated on Day 1 (left panel). ns: not significant, two‐way anova followed by Sidak's post hoc test. The recognition index of a novel object C was calculated for comparison on Day 2. *p < 0.05, **p < 0.01, Unpaired *t*‐test. (B, C) In T maze (B) and Y maze (C) tests, spontaneous alternation triplet percentage was calculated for comparison. ns: not significant, *p < 0.05, **p < 0.01, unpaired *t*‐test. *n* = 10 AAV‐Control female mice, *n* = 11 AAV‐RAB39B female mice, *n* = 11 AAV‐Control male mice, and *n* = 10 AAV‐RAB39B male mice. Data represent mean ± SEM.

In novel object recognition tests, RAB39B‐overexpressing mice and controls showed no preference for two identical objects (A and B) on the first day of test. However, compared with controls, mice with neuronal overexpression of RAB39B showed markedly reduced preference for the new object compared with the familiar object B when object A was replaced with a new object on the second day. These differences led to significantly lower object recognition index in RAB39B‐overexpressing mice than in control mice (Figure 2A). When different sexes were separated for comparison, both male and female mice with neuronal overexpression of RAB39B also had lower object recognition index than respective controls (Figure 2A). These results indicate that neuronal overexpression of RAB39B impairs the recognition memory of mice.

In both T and Y maze tests, mice with neuronal overexpression of RAB39B displayed comparable numbers of arm visits to but significantly lower spontaneous alternations than control mice (Figures 2B,C and S3D,E). When different sexes were separated for comparison, female RAB39B‐overexpressing mice also displayed significantly lower spontaneous alternations than controls in both T and Y maze test; and male RAB39B‐overexpressing mice also showed significantly reduced spontaneous alternations in the T maze test and a trend of reduction (though not significant) in the Y maze test compared with controls (Figure 2B,C). These results suggest that neuronal overexpression of RAB39B impairs short‐term working memory in mice.

However, in Morris water maze tests, neither male nor female mice with neuronal overexpression of RAB39B showed differences from respective controls in the escape latency to the platform during the training phase and in the time spent exploring the platform quadrant during the testing phase (Figure S4A–G). In addition, the freezing percentage of both male and female RAB39B‐overexpressing mice was comparable to that of respective controls in fear conditioning tests (Figure S4H–J). These results indicate that neuronal overexpression of RAB39B has no effect on learning and memory associated with space, context and cue in mice.

**FIGURE 3:**
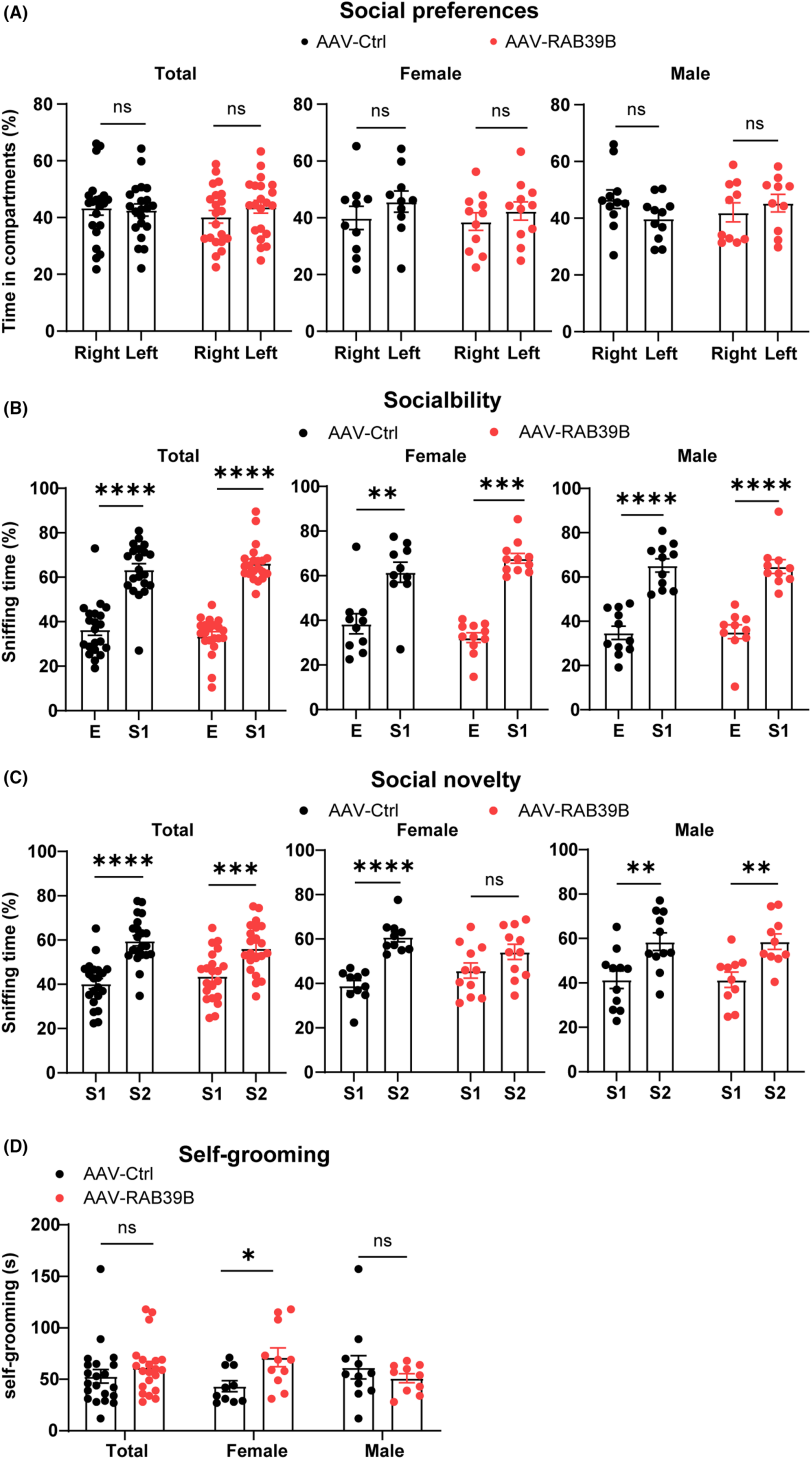
Neuronal overexpression of RAB39B leads to autism‐like behaviours in female mice. (A–C) In three‐chamber social interaction tests, mice were analysed for their time spent in exploring left and right chambers (A). Sociability was studied by comparing interactions with a stranger mouse (S1) and with an empty (E) cage (B). Social novelty was assessed by comparing interactions with the familiar mouse (S1) and with a novel stranger mouse (S2) (C). **p < 0.01, ***p < 0.001, ****p < 0.0001, two‐way anova followed by Sidak's post hoc test. (D) RAB39B‐overexpressing mice and controls were compared for their time spent on self‐grooming. ns: not significant, *p < 0.05, unpaired *t*‐test. *n* = 10 AAV‐Control female mice, *n* = 11 AAV‐RAB39B female mice, *n* = 11 AAV‐Control male mice, and *n* = 10 AAV‐RAB39B male mice. Data represent mean ± SEM.

We also studied whether neuronal overexpression of RAB39B affects social‐related activities. In marble‐burying test, neither male nor female mice with neuronal overexpression of RAB39B exhibited differences from respective controls (Figure S5). In three‐chamber social interaction tests, none of tested mice demonstrated preference for either the left or the right chamber during the adaptation phase (Figure 3A). During the testing phase, both male and female RAB39B‐overexpressing mice and respective controls spent more time interacting with a stranger mouse (Stranger 1, S1) than exploring an empty cage (E) (Figure 3B). Total and male RAB39B‐overexpressing mice also spent significantly more time interacting with a novel stranger mouse (Stranger 2, S2) than with the familiar mouse S1 just like what controls behaved. However, female RAB39B‐overexpressing mice showed no difference to controls in interacting with mouse S2 and the familiar mouse S1 (Figure 3C). Moreover, in self‐grooming tests, female but not male RAB39B‐overexpressing mice spent significantly more time grooming than respective control mice (Figure 3D). Together, these results imply that neuronal overexpression of RAB39B interferes with social novelty but not social preference and causes repetitive grooming behaviour in female mice.

### 3.2 Neuronal overexpression of RAB39B reduces synaptic transmission in female mice

**FIGURE 4:**
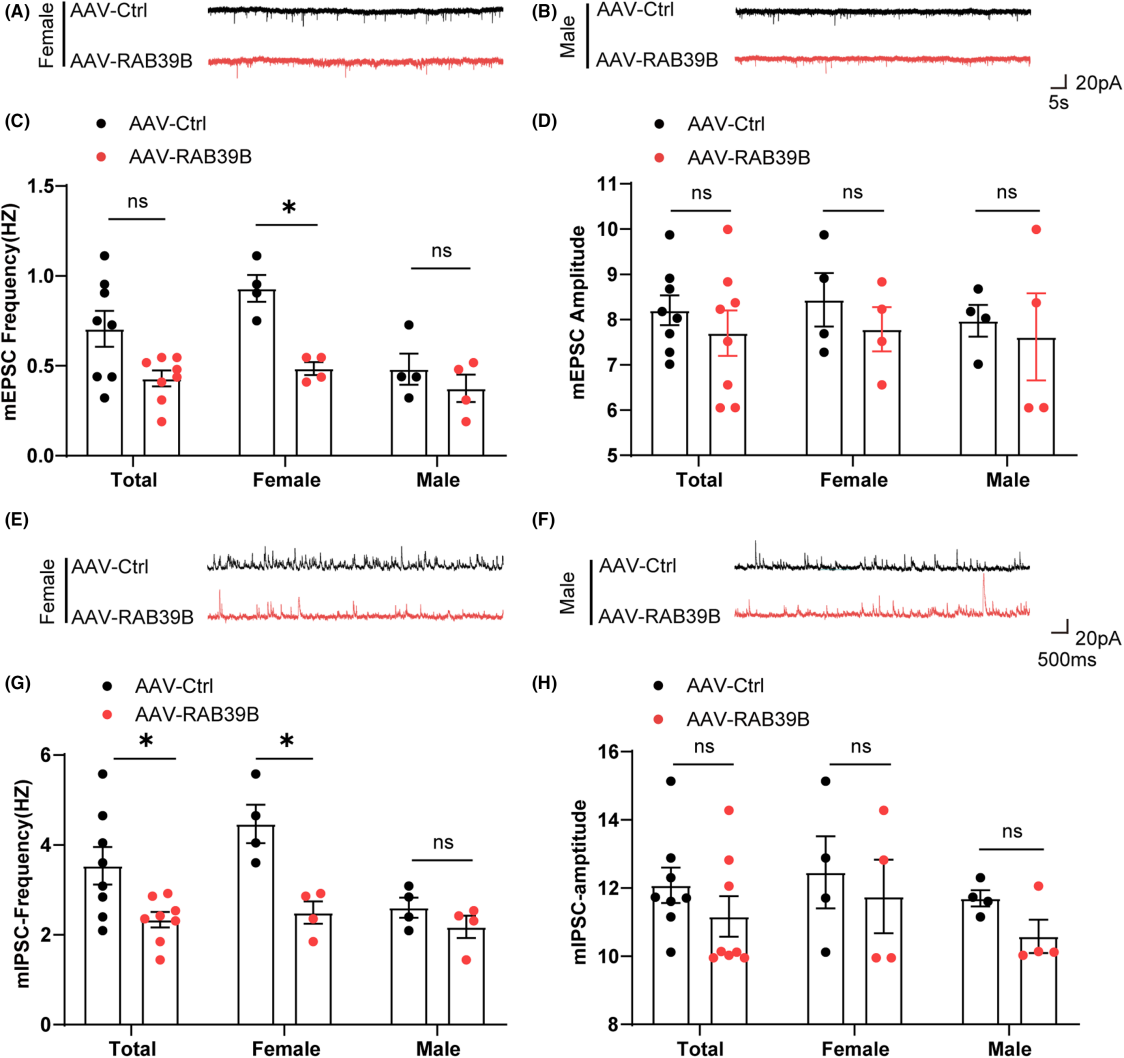
Neuronal overexpression of RAB39B reduces synaptic transmission in female mice. (A and B) Representative traces of mEPSCs in female (A) and male mice (B). (C and D) Quantitative analysis of the frequency (C) and amplitude (D) of mEPSCs. (E and F) Representative traces of mIPSCs in female (E) and male mice (F). (G and H) Quantitative analysis of the frequency (E) and amplitude (F) of mIPSCs. *n* = 4 female or male mice per group, average of 3–5 neurons from each mouse. Data represent mean ± SEM, ns: not significant, * *p* < 0.05, Mann–Whitney test.

Our previous study demonstrates that loss of RAB39B leads to excitatory synaptic dysfunction.^12^ Given the impairment of working and cognitive memory in mice with RAB39B overexpression, we also explored whether neuronal overexpression of RAB39B affects synaptic function. We found that the frequency but not the amplitude of miniature excitatory postsynaptic currents (mEPSCs) was significantly decreased in female RAB39B‐overexpressing mice compared with control mice (Figure 4A,C,D). In addition, the frequency but not the amplitude of miniature inhibitory postsynaptic currents (mIPSCs) was also significantly decreased in female RAB39B‐overexpressing mice compared with control mice (Figure 4E,G,H). However, neither mEPSCs nor mIPSCs were altered in male RAB39B‐overexpressing mice compared with controls (Figure 4B–D,F–H). When both males and females were combined, only the frequency of mIPSCs was significantly decreased in RAB39B‐overexpressing mice compared with controls (Figure 4G). Together, neuronal overexpression of RAB39B impairs synaptic transmission in female mice.

### 3.3 Neuronal overexpression of RAB39B decreases dendritic arborizations

**FIGURE 5:**
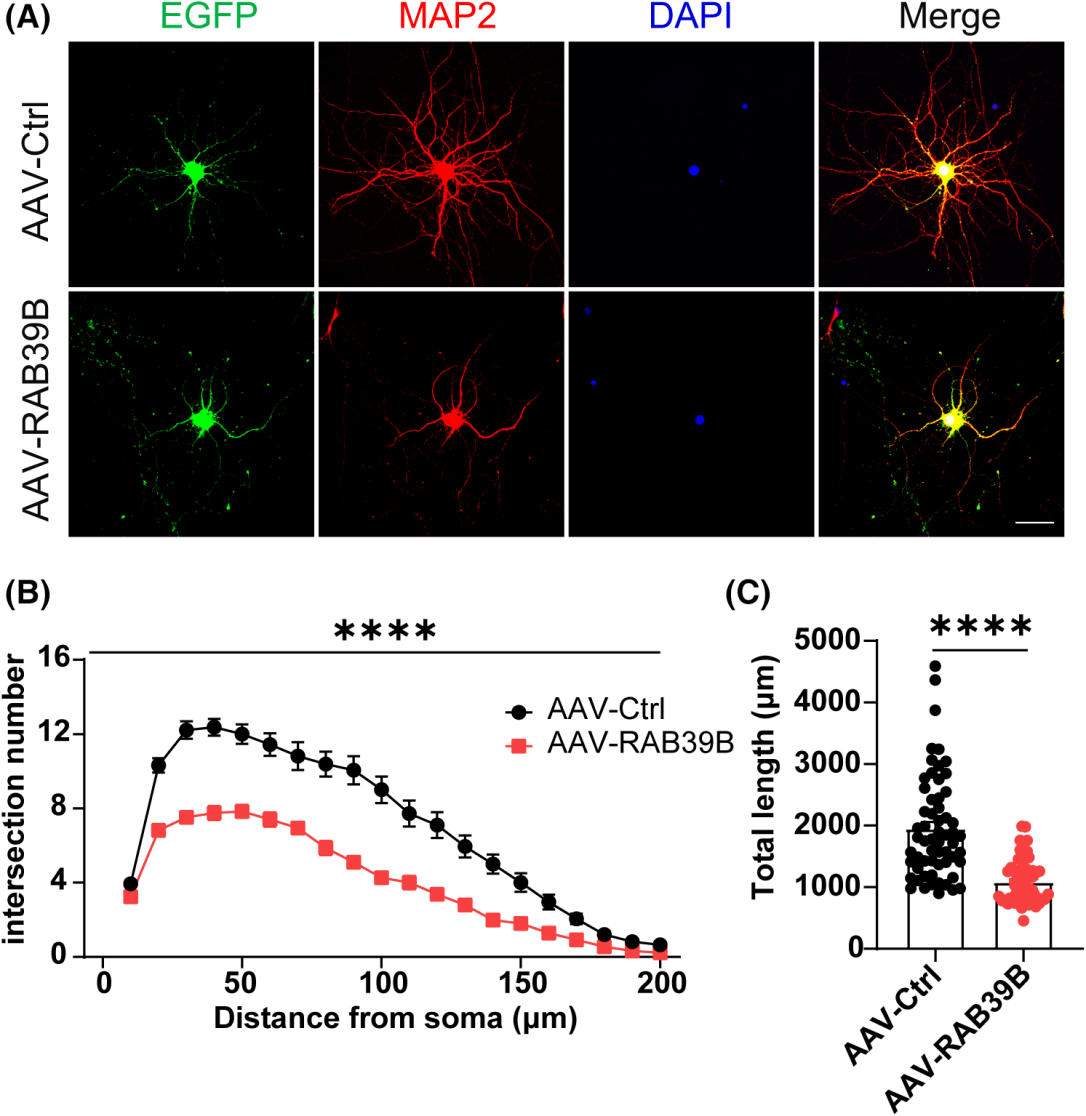
Neuronal overexpression of RAB39B decreases dendritic arborization. (A) Representative images of MAP2 (red) and DAPI (blue) immunostaining of primary hippocampal neurons at DIV 12 infected with AAV‐Control or AAV‐RAB39B (green). Scale bars, 50 μm. (B) Sholl analysis of dendritic arborization stained with MAP2 in (A). *n* = 60 neurons from three independent preparations per group. Two‐way anova followed by Sidak's post hoc test. (C) Quantitative analysis of total length of neurites in (A). *n* = 60 neurons from three independent preparations per group, Mann–Whitney test. All data represent mean ± SEM, ****p < 0.0001.

Many neurodevelopmental disorders are accompanied by impaired dendritic development including dendritic arbour morphological features.^30^, ^31^ We analysed the morphology of neurons in vitro by immunofluorescence staining. Primary hippocampal neurons derived from wild‐type mice were infected with AAVs expressing RAB39B or EGFP controls. We found that neuronal overexpression of RAB39B resulted in decreased dendritic complexity and total dendritic length compared with controls (Figure 5A–C).

However, immunoblotting analysis revealed that total amounts of presynaptic proteins (including VGLUT1, Synapsin 1 and Synaptophysin) and postsynaptic proteins and excitatory receptor subunits (including GluA1, GluA2, GluN1, GluN2A, GluN2B and PSD95) in the hippocampal region of RAB39B‐overexpressing mice were not different from those of control mice (Figure S6A,B). Moreover, neuronal overexpression of RAB39B had no effect on synaptosome and PSD distributions of these detected proteins (Figure S6C,D).

### 3.4 Neuronal overexpression of RAB39B alters autophagy

**FIGURE 6:**
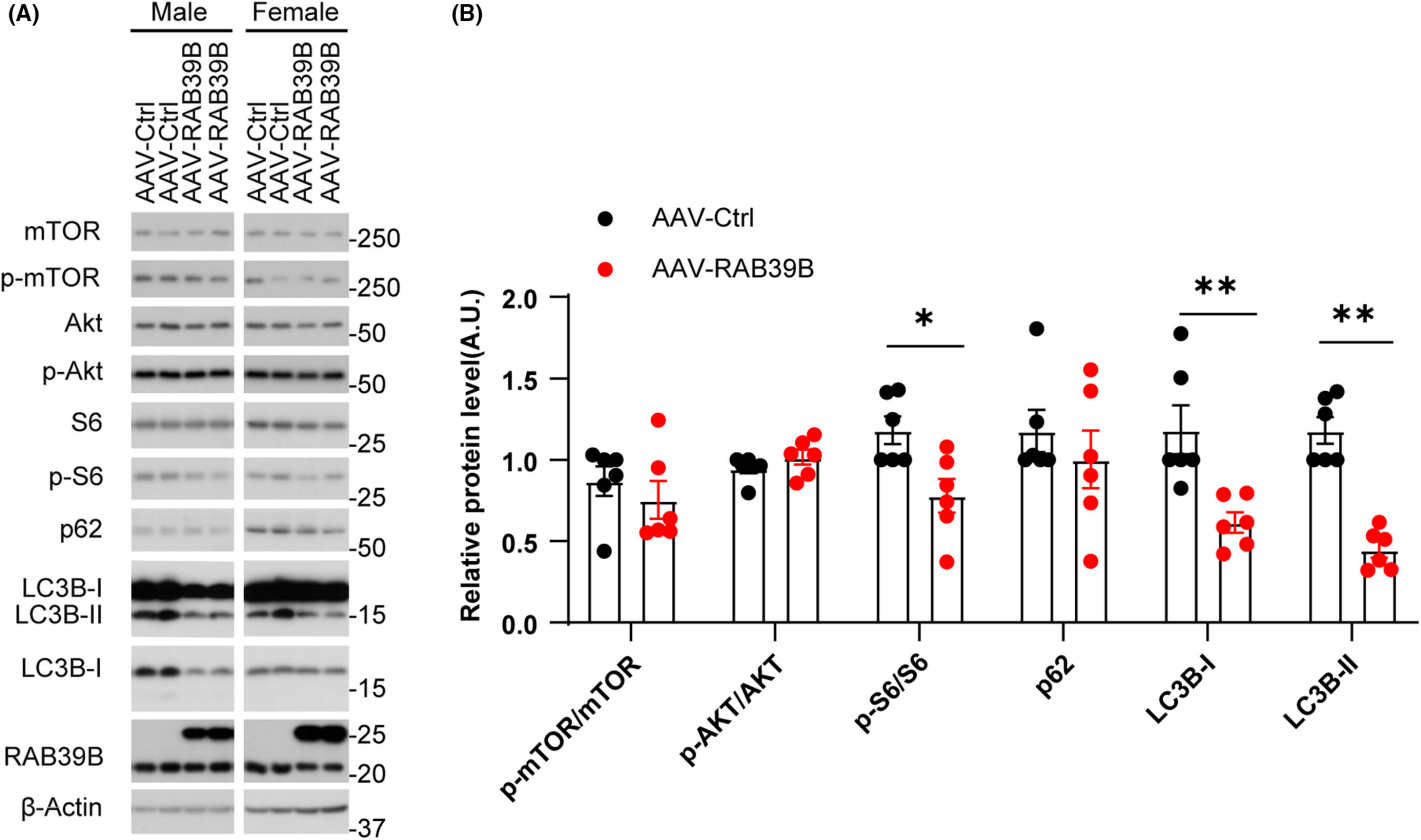
Neuronal overexpression of RAB39B alters autophagy. (A) Western blotting of proteins in the hippocampal region of AAV‐Control and AAV‐RAB39B mice. (B) Protein levels were quantified and normalized to those of β‐actin for comparison. Data represent mean ± SEM, *n* = 6 mice for each group, *p < 0.05, **p < 0.01, Mann–Whitney test.

Previous studies have found that RAB39B deficiency promotes the PI3K‐AKT–mTOR signalling pathway and impairs autophagy.^12^, ^13^ Consistently, we found that levels of phosphorylated S6 and LC3B were significantly decreased in the hippocampal region of RAB39B‐overexpressing mice compared with those of controls (Figure 6A,B), confirming that RAB39B participates in autophagy.

## 4 DISCUSSION

Since the first description of int22h1/int22h2‐mediated Xq28 duplication syndrome in 2011, a total of 35 cases have been reported in the literature.^6^, ^17^ Common clinical manifestations of this XLID syndrome include intellectual disability, neurobehavioural abnormalities and nonspecific facial dysmorphic features.^16^ The reported duplicated fragments contain at most 6 genes (*FUNDC2*, *MTCP1*, *BRCC3*, *VBP1*, *RAB39B* and *CLIC2*), among which *RAB39B* and *CLIC2* are presumed to be responsible for disease phenotypes because the two genes are included in the smallest duplicated region in patients with int22h1/int22h2‐mediated Xq28 duplication found so far.^18^, ^19^, ^20^, ^21^, ^32^ Indirect supporting evidence comes from the findings that loss‐of‐function mutations in the two genes are also associated with XLID.^9^, ^10^, ^33^ However, whether excessive RAB39B really leads to XLID has never been verified in animal models. Our previous work has shown that loss of RAB39B impairs autophagy and synaptic structure and function, resulting in defective learning and memory in mice.^12^ In the current study, we have further demonstrated that neuronal overexpression of RAB39B also impairs neuronal development and synaptic transmission, thereby compromising certain cognitive and social behaviours with probable sex differences in mice.

We injected AAVs expressing RAB39B into bilateral ventricles of wild‐type postpartum day 0 (P0) mice and observed recognition memory impairment in both male and female mice at 2 months of age. This result supports that an increased dosage of RAB39B may be the cause of intellectual disability phenotypes in patients. Male patients with int22h1/int22h2‐mediated Xq28 duplication syndrome show mild‐to‐moderate intellectual disability, accompanied by a variety of neurobehavioural manifestations such ASDs, anxiety, irritability and social disabilities, whereas female patients usually exhibit lighter disease symptoms than males.^6^, ^16^ Such a gender difference may be attributed to genetic factors, since affected males are hemizygotic but affected females are mostly heterozygous and may undergo skewed inactivation of the X chromosome.^15^, ^20^, ^34^ However, in the present study we found that neuronal overexpression of RAB39B seems to cause more several disease manifestations in female than male mice, as female but not male RAB39B‐overexpressing mice exhibited social novelty defect and repetitive grooming behaviour. The reason for such a sex‐related phenotype discrepancy between humans and mice is not clear. Since the expression of exogenous RAB39B is much stronger than that of endogenous RAB39B, one possibility is that females are more susceptible than males to overly high expression of RAB39B, which dose cannot be achieved in human patients. Alternatively, since CLIC2 is also excessively expressed in human patients but not in our animal models used here, extra CLIC2 may have stronger effect on causing disease phenotypes than RAB39B in male patients. All these possibilities deserve further scrutiny.

Abnormality of neuronal dendrite growth and spine development may jeopardize neuronal functions and leads to a series of nervous system diseases such as intellectual disability and ASDs.^30^, ^35^ Both knockdown and overexpression of RAB39B have been found to cause a decrease in dendritic arborizations of primary hippocampal neurons.^9^, ^21^ Neuronal branching and numbers of presynaptic terminals decreased after overexpression of RAB39B in primary hippocampal neurons in vitro.^21^ Here, we also confirmed that neuronal overexpression of RAB39B significantly attenuated dendritic arborizations of neurons in vitro. Interestingly, neuronal overexpression of RAB39B only impaired mEPSC and mIPSC frequencies in CA1 neurons of female but not male mice. This is in parallel to behavioural tests, showing that RAB39B overexpression caused more disease phenotypes in female than male mice. One possible explanation for this female‐specific synaptic dysfunction is that certain intrinsic factors in males but not females protect compromised neurons by RAB39B overexpression against functional impairment; and this deserves further scrutiny.

Dendritic spines are postsynaptic sites of most excitatory glutamatergic synapses in the mammalian brain and contain essential molecular components related to postsynaptic signalling and plasticity.^36^, ^37^, ^38^ RAB39B has been shown to affect maturation and trafficking of AMPA receptor subunits.^14^, ^39^ We previously also found NMDA receptor reductions in the PSD of *Rab39b* knockout mice.^12^ However, here we found that none of the detected postsynaptic proteins including AMPA and NMDA receptor subunits were altered upon RAB39B overexpression, and the discrepancy may be attributed to the different mouse lines used. Moreover, we only generally analysed the total levels and PSD distribution of the postsynaptic proteins fractionated from mouse hippocampal region, and the immunofluorescence staining should be used for further scrutiny of the subcellular localization of these proteins. Since only mEPSC and mIPSC frequencies but not their amplitudes were altered upon neuronal overexpression of RAB39B, excessive RAB39B may function preferentially in presynaptic regions. Although levels of VGLUT1, Synapsin 1 and Synaptophysin were unaltered upon neuronal overexpression of RAB39B, further study on additional presynaptic proteins may help clarifying the role of excessive RAB39B in presynaptic regions.

It has been reported that a loss of RAB39B promotes PI3K‐AKT–mTOR pathway and impairs autophagy.^12^, ^13^ Herein, we also found significantly decreased phosphorylated S6 and LC3B‐II in the hippocampal region of RAB39B‐overexpressing mice. We previously also found that the treatment of mTOR inhibitor rapamycin improved novel object recognition memory and rescued LTP deficits in *Rab39b* KO mice, indicating that rapamycin might alleviate XLID symptoms.^12^ However, the contribution of RAB39B to XLID has yet to be further confirmed.

In summary, our results demonstrate that neuronal overexpression of RAB39B impairs normal development of neurons and leads to synaptic dysfunction and certain behavioural defects in mice. These findings indicate the contribution of increased dosage of RAB39B to the pathogenesis of XLID associated with Xq28 duplications. Moreover, since neuronal overexpression of RAB39B alters autophagy, targeting autophagy may provide novel strategies for disease treatment.

## AUTHOR CONTRIBUTIONS

**Zijie Wang:** Data curation (equal); formal analysis (equal); investigation (equal); validation (equal); writing – original draft (equal). **Mengxi Niu:** Data curation (equal); formal analysis (equal); investigation (equal); validation (equal). **Naizhen Zheng:** Data curation (equal); investigation (equal). **Jian Meng:** Data curation (equal); investigation (equal). **Yiru Jiang:** Data curation (equal); investigation (equal). **Dingting Yang:** Data curation (equal); investigation (equal). **Peijie Yao:** Data curation (equal); investigation (equal). **Tingting Yao:** Data curation (equal); investigation (equal). **Hong Luo:** Project administration (supporting); visualization (supporting). **Huaxi Xu:** Project administration (supporting); supervision (supporting). **Yunlong Ge:** Project administration (supporting); supervision (supporting). **Yun‐Wu Zhang:** Conceptualization (equal); funding acquisition (lead); methodology (supporting); project administration (equal); resources (supporting); supervision (equal); writing – original draft (equal); writing – review and editing (equal). **Xian Zhang:** Conceptualization (supporting); methodology (supporting); project administration (equal); resources (supporting); supervision (equal); writing – original draft (equal); writing – review and editing (equal).

## CONFLICT OF INTEREST STATEMENT

The authors declare that the research was conducted in the absence of any commercial or financial relationships that could be construed as a potential conflict of interest.

## Supporting information


**Figures S1.** Supplementary material

## Data Availability

The data that support the findings of this study are available from the corresponding author upon reasonable request.
