# Supplementary material for: Increased level of RAB39B leads to neuronal dysfunction and behavioural changes in mice
Source: J Cell Mol Med. 2023 Mar 28;27(9):1214–26. doi: 10.1111/jcmm.17704 (PMC10148058; doi:10.1111/jcmm.17704)
Supplement: Supplementary file 1 — Figures S1. Supplementary material [file JCMM-27-1214-s001.docx]

**SUPPLEMENTARY FIGURES**


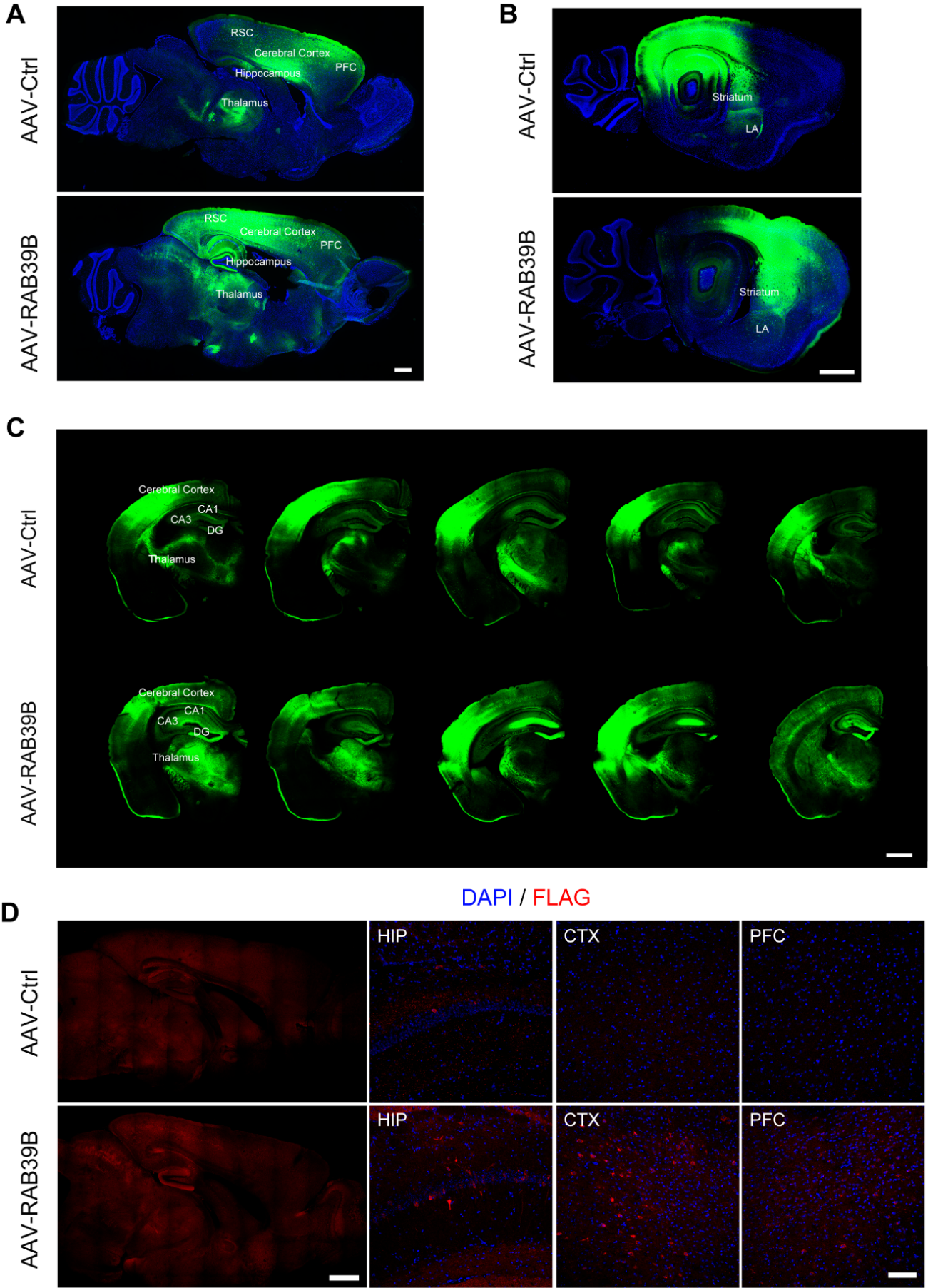


**SUPPLEMENTARY FIGURE 1** Neuronal overexpression of RAB39B in the brain of mice. **(A-C)** Representative sagittal **(A, B)** and coronal **(C)** images of EGFP expression in mice with AAV-Ctrl and AAV-RAB39B injection. Scale bars, 1000 μm. RSC: retrosplenial cortex; PFC: prefrontal cortex; M1: primary motor cortex; M2: secondary motor cortex; mPFC: medial prefrontal cortex; LA: lateral nucleus of the amygdala. **(D)** Representative immunofluorescence images of sagittal brain and partial regions stained with an anti-FLAG antibody in mice with AAV-Control and AAV-RAB39B injection. Scale bars, 1000 μm for entire brain images and 100 μm for enlarged images. HIP: hippocampus; CTX: cerebral cortex; PFC: prefrontal cortex.


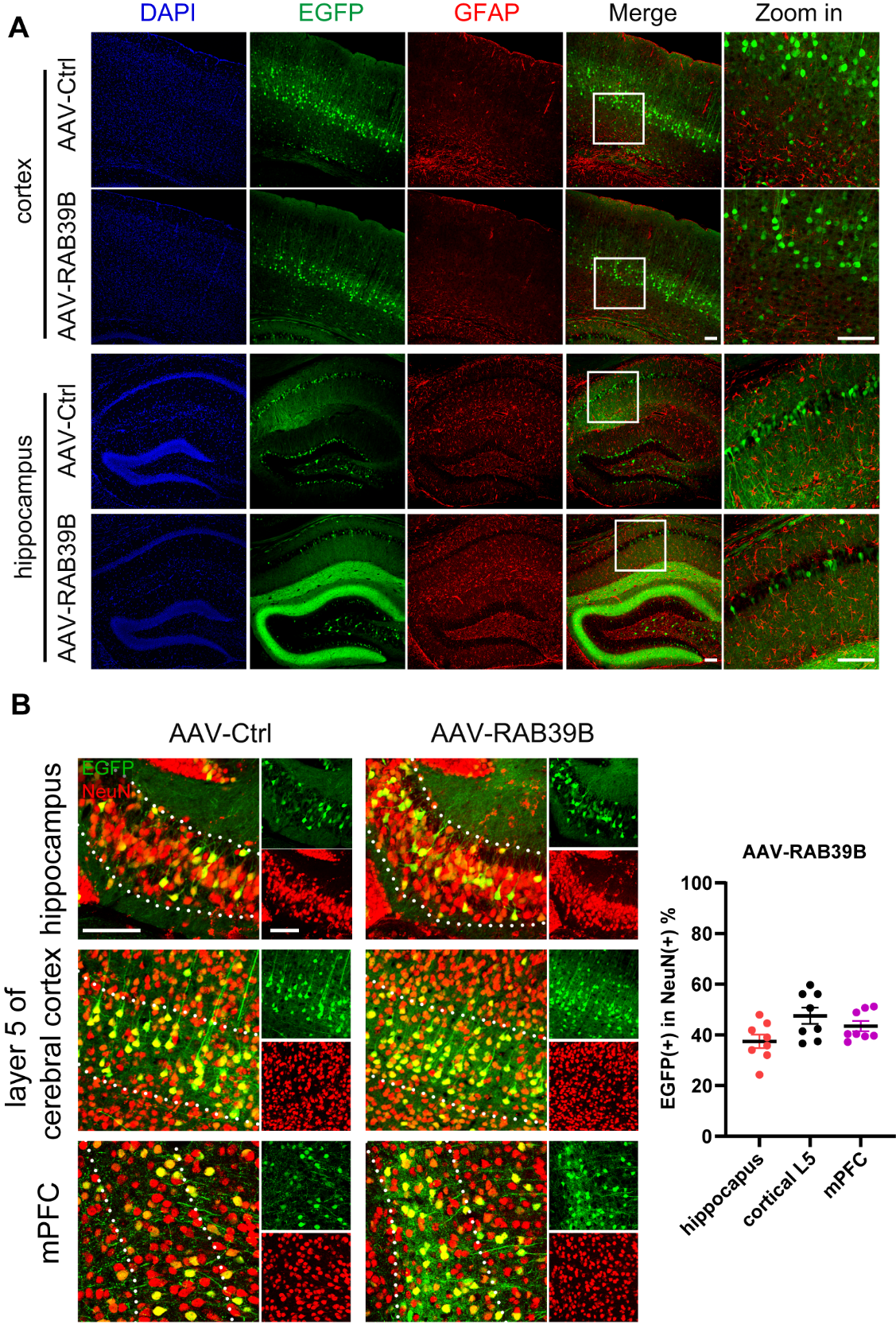


**SUPPLEMENTARY FIGURE 2** EGFP expression predominantly colocalizes with NeuN- but not GFAP-positive cells. **(A)** Brain sections from mice injected with AAV-Control and AAV-RAB39B (represented by EGFP in green) were subjected to immunostaining with GFAP (red) and staining with DAPI (blue). Representative images of hippocampus and cortex are shown. Scale bars, 50 μm. **(B)** Brain sections from mice injected with AAV-Control and AAV-RAB39B (represented by EGFP in green) were subjected to immunostaining with NeuN (red). Representative images of hippocampus CA3 region, layer 5 of cerebral cortex, and medial prefrontal cortex (mPFC) are shown. Scale bars, 50 μm. The percentages of EGFP-positive cells in all NeuN-positive cells in different brain regions were counted for comparison. One brain section from each mouse showing the same layer of the hippocampus, cerebral cortex and mPFC was used for co-localization analysis, n = 8 brain sections from 8 different mice.


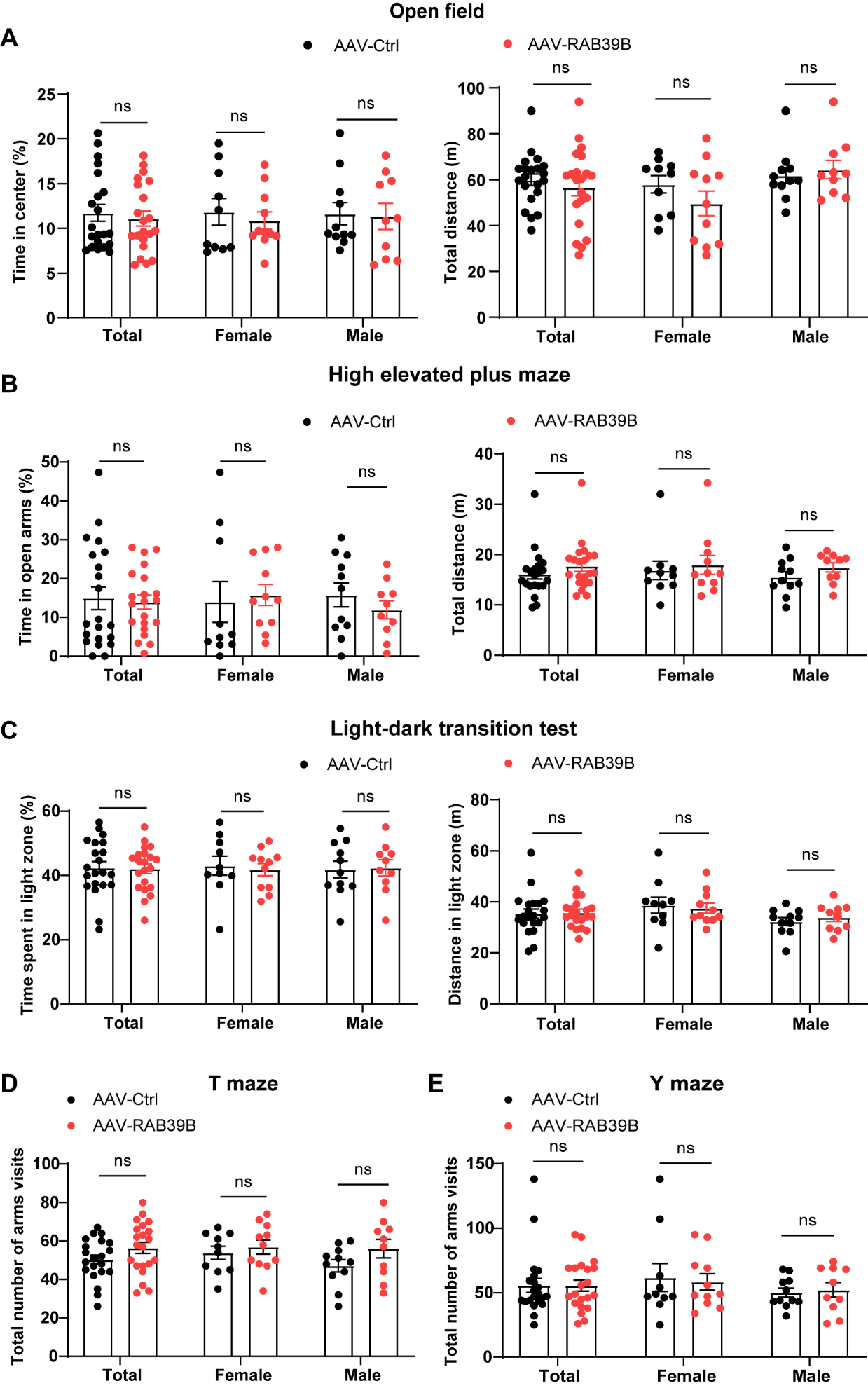


**SUPPLEMENTARY FIGURE 3** Neuronal overexpression of RAB39B does not affect anxiety in mice. **(A)** The time spent in the center and total travel distance of AAV-Control and AAV-RAB39B mice were recorded for analysis in open field tests. **(B)** The time spent in open arms and total travel distance of AAV-Control and AAV-RAB39B mice were recorded for analysis in high elevated plus maze tests. **(C)** The time spent and total distance traveled in the light zone of AAV-Control and AAV-RAB39B mice were recorded for analysis in light-dark transition tests. **(D-E)** In T maze **(D)** and Y maze **(E)** tests, the total numbers of arms visits were measured for comparison. n = 10 AAV-Control female mice, n = 11 AAV-RAB39B female mice, n = 11 AAV-Control male mice, and n = 10 AAV-RAB39B male mice. Data represent mean ± SEM, ns: not significant, Unpaired t-test.


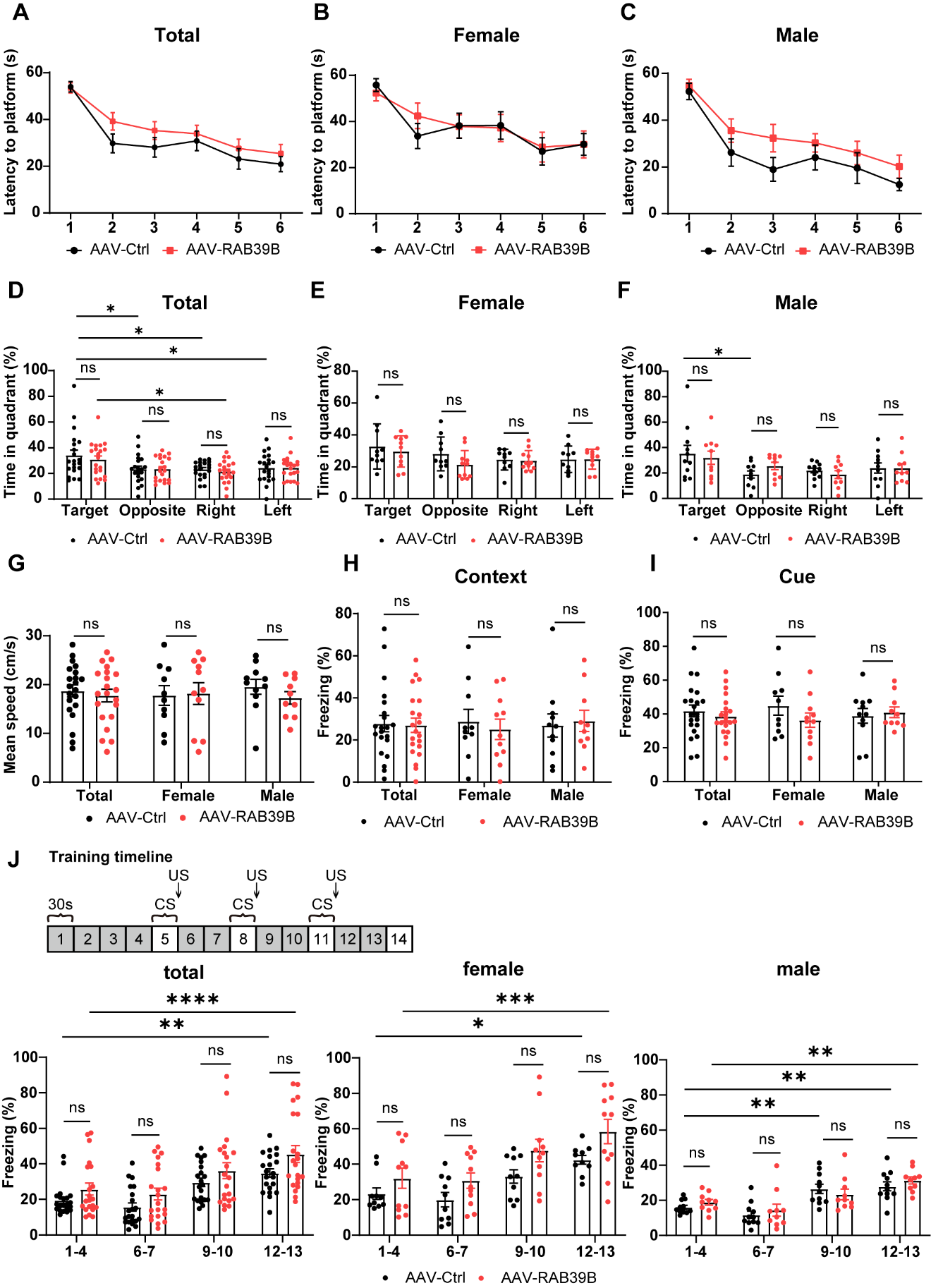


**SUPPLEMENTARY FIGURE 4** Neuronal overexpression of RAB39B does not affect certain learning and memory in mice. **(A-G)** Escape latency of total **(A)**, female **(B)**, and male **(C)** mice was recorded in Morris Water Maze tests within a 6-day training period. On the 7th day, total **(D)**, female **(E)**, and male **(F)** mice were assayed for their time spent in the target and the other three quadrants. Mean speed **(G)** of mice were recorded at the 7th day. ns: not significant, **p* < 0.05, Two-way ANOVA followed by Sidak's post hoc test. **(H-I)** In fear conditioning tests, mice were trained and analyzed for freezing responses under both contextual **(H)** and cued **(I)** tests. Freezing response is expressed as a percentage of the total duration. ns: not significant, Unpaired t-test. **(J)** A schematic diagram shows a training timeline of 3 times CS-US pairing implementations. Freezing response before the occurrence of CS-US paring and after each stimulus. ns: not significant, **p* < 0.05, ***p* < 0.01, ****p* < 0.001, *****p* < 0.0001, Two-way ANOVA followed by Sidak's post hoc test. n = 10 AAV-Control female mice, n = 11 AAV-RAB39B female mice, n = 11 AAV-Control male mice, and n = 10 AAV-RAB39B male mice. Data represent mean ± SEM.


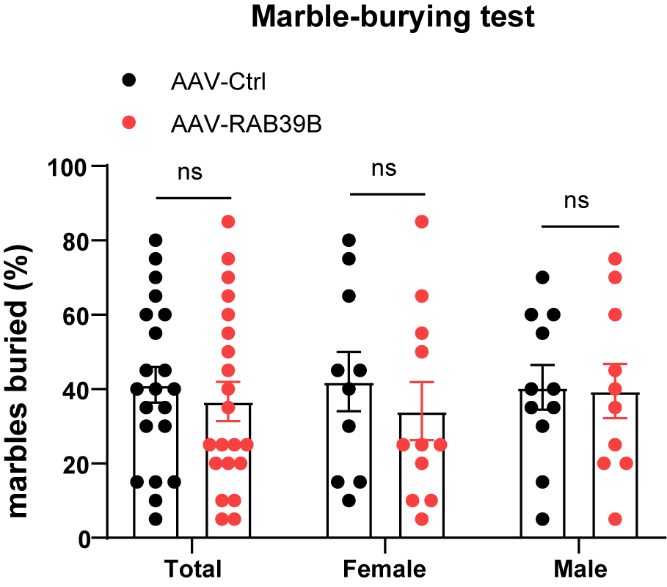


**SUPPLEMENTARY FIGURE** **5** Neuronal overexpression of RAB39B does not affect marble-burying behavior. Percentage of marbles buried was analyzed for comparison. n = 10 AAV-Control female mice, n = 11 AAV-RAB39B female mice, n = 11 AAV-Control male mice, and n = 10 AAV-RAB39B male mice. Data represent mean ± SEM, ns: not significant, Unpaired t-test.


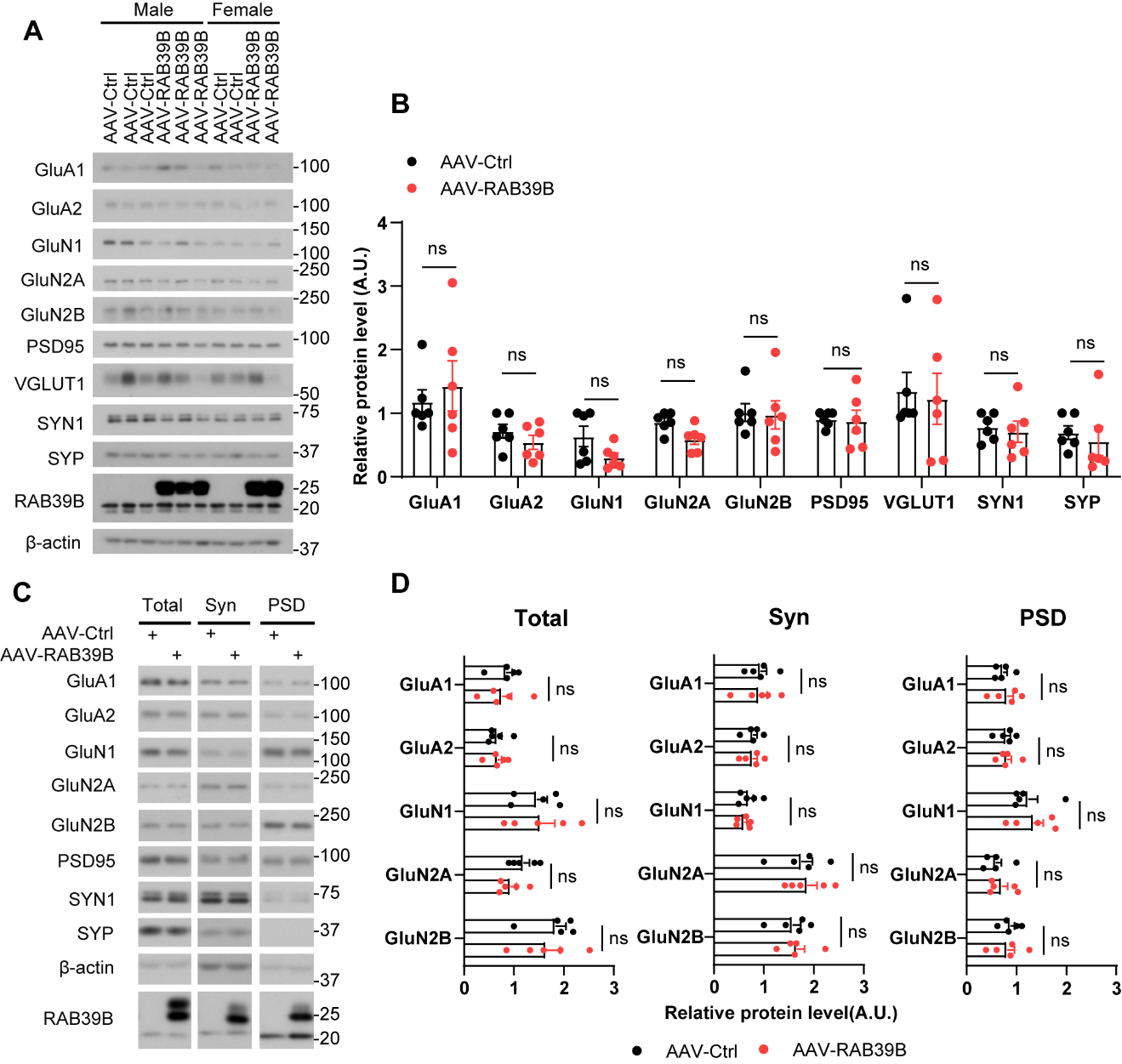


**SUPPLEMENTARY FIGURE 6** Neuronal overexpression of RAB39B has no effect on total levels and PSD distribution of synaptic proteins. **(A)** Western blotting for proteins in the hippocampal region of AAV-Control and AAV-RAB39B mice. **(B)** Protein levels were quantified and normalized to those of β-actin for comparison. n = 6 mice for each group. **(C)** Western blotting for proteins in total, synaptosome (Syn) and PSD fractions derived from AAV-Control and AAV-RAB39B mouse brain. **(D)** Protein levels were quantified and normalized to those of β-actin for comparison. n = 5 mice for each group. Data represent mean ± SEM, ns: not significant, Mann-Whitney test.
